# Supplementary material for: Achieving sub-pm wavelength regression via minimum-phase in a single-stream photonic IC
Source: Nat Commun. 2026 Mar 25;17:4464. doi: 10.1038/s41467-026-71087-7 (PMC13187246; doi:10.1038/s41467-026-71087-7)
Supplement: Supplementary file 1 — Supplementary Information [file 41467_2026_71087_MOESM1_ESM.pdf]

Achieving Sub-pm Wavelength Regression via Minimum-Phase in a Single-Stream Photonic IC: Supplemental

Hector A. Rubio Rivera[1,*], Lilian Neim[1], Venkatesh Deendayalan[1], Stefan Preble[1]
[1]Electrical and Microelectronic Engineering, Rochester Institute of Technology, NY, USA
[*]Corresponding author: hr8392@rit.edu

I. Minimum-Phase Systems in Photonic Design

A. The Gain-Phase Relationship in Minimum-Phase Systems

The Hilbert transform relates the real and imaginary parts of the frequency response of a causal system that is also analytic in the upper half-plane of ω [1]. However, we are interested in finding how a given system's phase and magnitude may also be related. We start by expressing the system $\hat{H}(\omega)$ as

$$\hat{H}(\omega) = \log\left(H(\omega)\right) = \log\left(|H(\omega)|\right) + j\theta(\omega) \tag{1}$$

where $|H(\omega)|$ and $\theta(\omega)$ are the phase and magnitude of a causal system $H(\omega)$. By taking the natural log of $H(\omega)$, we can define a system with real and imaginary parts as the phase and magnitude of $H(\omega)$.

We can assume, without loss of generality, that the transfer function $\hat{H}(\omega)$ can be expressed as a ratio of two polynomials in the Laplace domain, such as

$$\hat{H}(s) = \frac{N(s)}{D(s)} \tag{2}$$

where the zeros are the s values that cause $N(s)$ to be zero and the poles are the s values that cause $D(s)$ to be zero. Since $s = j\omega$, we can understand the s-domain as a 90-degree rotation from the ω-domain [1]. This causes the ω upper half plane to become the s right half plane. Therefore, if $\hat{H}(s)$ is analytic on the right half plane, the real and imaginary parts of $\hat{H}(\omega)$ are correlated by the Hilbert transform, which means that the phase and magnitude of $H(\omega)$ are also correlated by the Hilbert transform. We can take the derivative of $\hat{H}(s)$ with respect to s such as [2]

$$\frac{d\hat{H}(s)}{ds} = \frac{1}{N}\frac{dN}{ds} + \frac{1}{D}\frac{dD}{ds} \tag{3}$$

which, is only true if $\hat{H}(s)$ is analytic everywhere except on the zeros and poles locations of $H(\omega)$. As long as the zeros and poles of the original system $H(\omega)$ are on ω lower half plane or in the s left-hand plane, the phase and magnitude are correlated by the Hilbert transform. As a result, if a system's transfer function (zeros of N) and its inverse (zeros of D) are both causal the system follows Kramers-Kronig relationships and is otherwise known as a minimum-phase system.

Since the real and imaginary parts of $\hat{H}(\omega)$ are related, we can express these as a Hilbert transform pair similar to the real and imaginary parts of $H(\omega)$. Therefore, the magnitude and phase of $H(\omega)$ are related by the pair of equations

$$\theta(\omega) = \frac{1}{\pi}\int_{-\infty}^{\infty} \frac{\log\left(|H(\omega')|\right)}{\omega - \omega'}\, d\omega' \tag{4}$$

$$\log\left(H(\omega)\right) = \frac{1}{\pi}\int_{-\infty}^{\infty} \frac{\theta(\omega')}{\omega - \omega'}\, d\omega' \tag{5}$$

which means that we can retrieve the phase of $H(\omega)$ through a Hilbert transformation of $\log\left(|H(\omega)|\right)$.

B. Minimum-Phase Systems in Photonic Design

As shown in Figure S1, we can take advantage of the photonic integrated circuit platform to divide the system into two paths easily by using beam splitters. In doing so, the system transfer function is described by Equation (6).

$$H(\omega) = \frac{Y}{X} = H_{ref}(\omega) + H_{cpu}(\omega) \tag{6}$$

where $H_{ref}(\omega)$ and $H_{cpu}(\omega)$ contain the circuit's frequency information dedicated to each path. Note that, in general, this circuit can be any photonic structure, $i.e.$ IIR filters such as micro-ring modulators, and FIR filter structures such as Mach-Zehnder interferometers. We examine $H(\omega)$ in this scenario to find the conditions that make $H(\omega)$ a minimum-phase system. $H(\omega)$ is a minimum phase system if the system and its inverse are causal [1]. Both $H_{ref}(\omega)$ and $H_{cpu}(\omega)$ are causal since they depend only on current or past values, $i.e.$ we will not get a response out of these systems if the input has not been excited. Therefore, $H(\omega)$ as the addition of two causal systems, is also causal. Looking at the inverse system,

$$\frac{1}{H(\omega)} = \frac{1}{H_{ref}(\omega) + H_{cpu}(\omega)} \tag{7}$$

we can perform the following factorization [3]

$$\frac{1}{H(\omega)} = \frac{1}{H_{ref}(\omega)} + \frac{1}{H_{ref}(\omega)} \frac{\frac{-H_{cpu}(\omega)}{H_{ref}(\omega)}}{1 - \frac{-H_{cpu}(\omega)}{H_{ref}(\omega)}} \tag{8}$$

noting that the last factor is the result of a geometric sum if and only if

$$\left| \frac{-H_{cpu}(\omega)}{H_{ref}(\omega)} \right| < 1 \tag{9}$$

therefore, Equation 8 can be expanded as a geometric series such as

$$\frac{1}{H(\omega)} = \frac{1}{H_{ref}(\omega)} + \frac{1}{H_{ref}(\omega)} \sum_{n=1}^{\infty} \left(\frac{-H_{cpu}(\omega)}{H_{ref}(\omega)} \right)^n \tag{10}$$

providing a sum of cascaded causal systems making the resulting system causal. The conclusion is that the power going into the $H_{ref}(\omega)$ part of the system has to be larger than the power going into $H_{cpu}(\omega)$. Consequently, this also ensures that $H_{ref}(\omega)$ contains the smallest delay within the system in a PIC context due to waveguide loss.

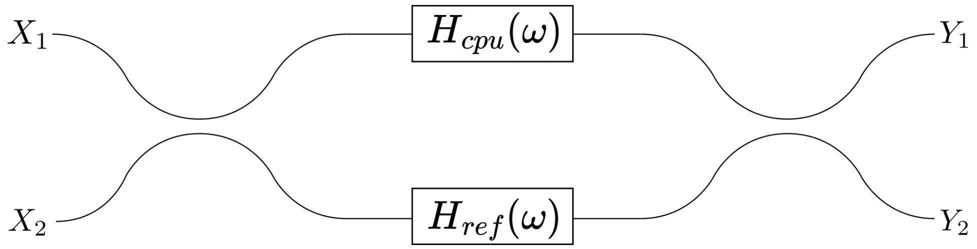

Figure S1. **Minimum-Phase Analysis in Photonic Integrated Circuits**. Photonic system structure partitioned for its analysis as a minimum-phase system.

II. Single ΔL: Calibration & Testing Algorithm

The calibration and testing algorithms used for the data presented in Figure 2 of the main paper are explained in detail in Fig. S2. The calibration process shown in Fig. S2(a) begins with the photodetected signal from a known laser and its logged wavelength values (e.g., a Keysight 8164B laser with "lambda logged" wavelength calibrated sweeps). Leveraging the MZI's minimum-phase design, the phase is retrieved via a Hilbert Transform and used to construct a complex-valued frequency response. The Fourier Transform of this response reveals a peak at $e^{i\beta\Delta L}$, which guides the design of a digital bandpass filter (h_{bpf}) centered at this peak. Applying the filter isolates the $\beta\Delta L$ phase, which is then unwrapped to build a linear phase-to-wavelength model.

The testing flow, shown in Fig. S2(b) for matched ΔL and Fig. S2(c) for mismatched ΔL, follows a similar process. The key difference is that instead of designing a new filter, the learned filter and model from calibration are applied to the complex-valued frequency response after phase retrieval. When ΔL values match, slopes align, and RMSE remains small; mismatched ΔL values lead to a substantial increase in RMSE. This indicates that regression results are driven by the circuit's physical properties rather than the filtering process. Figure 2(d) of the main paper summarizes the RMSE data for 49 circuits across two packages (P1, P2), each containing four circuits (note: PD output of K_4 was shorted).

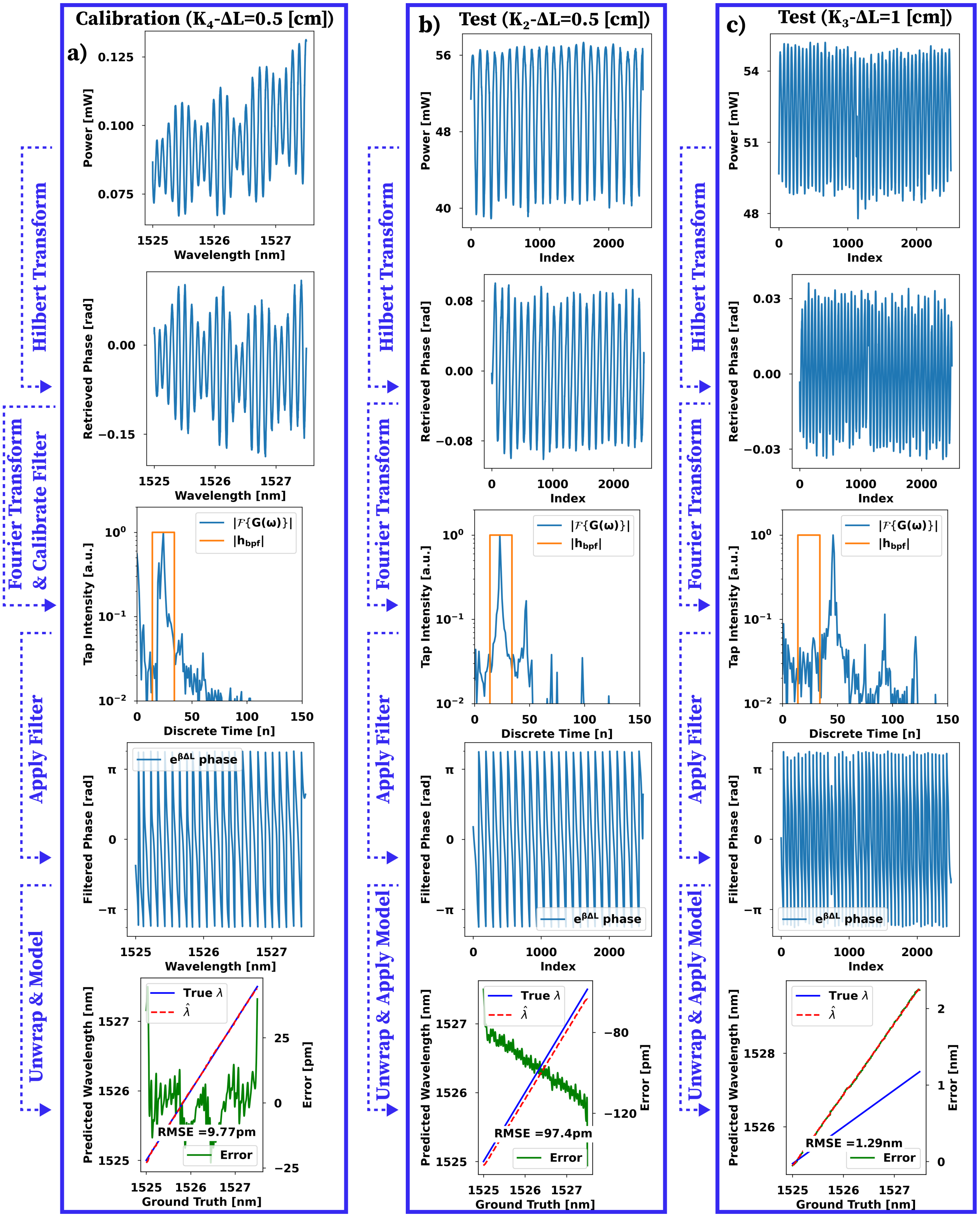

Figure S2. **Calibration and Testing Algorithm for Single ΔL Design (Section II.B)**. (a) The calibration process begins with the photodetected signal from a known laser source and its corresponding wavelength values. A Hilbert transform is applied to retrieve the phase, leveraging the MZI's minimum-phase property. This phase is used to construct a complex-valued frequency response, enabling the design of h_{bpf} centered at the peak ($e^{\beta \Delta L}$). The filter is then applied to isolate the $\beta\Delta$ phase, which is subsequently unwrapped to create a linear phase-to-wavelength model. (b) The testing algorithm applies the learned filter and model to the same ΔL as in calibration, and (c) to a different ΔL, demonstrating that regression results are driven by the circuit's physical properties rather than filter characteristics. This indicates that different ΔL values result in different slopes, causing an increase in RMSE. Figure 2(d) of the main paper summarizes the data for 49 circuits across two packages (P1, P2), each containing four circuits (note: PD output of K_4 was shorted).

III. Tap Extraction: Dispersion & Signal Reconstruction

Extracting the delay line information found in the Fourier transform depends on the proper design of the MZI circuit and handling of the dispersion. In addition, we show that reconstruction of the original $G(\omega)$ is possible when the Fourier transform information has been properly conditioned. Here, we show that the optical delay lines present in the 8-tap MZI circuit presented in the main article are improperly designed for accurate representation in the Fourier domain.

A. Dispersion

The wavelength model uses a Taylor expansion on the wavevector β. This results in an approximation that is only valid for a bandwidth (Δf) that has been properly modeled. We note that, for integrated photonic circuits, $\frac{d^2\beta}{df^2} \neq 0$. That is, the group index dispersion is not zero. As a result, we have a phase that is not linear in frequency/wavelength. This has profound implications for the Fourier transformation of $G(\omega)$. As shown in Figure S3, when we increase the wavelength bandwidth from 10 nm to 100 nm, the resulting Fourier transformation of $G(\omega)$ (red trace in Figure S3(b)) shows a high degree of Fresnel ripples as a consequence of the phase showing a quadratic dependence of frequency. Since $\Delta L2 \approx 2\Delta L1$ for the 4-tap MZI, the Fresnel ripples cause these two delay lines to merge into what appears to be a single tap. This causes improper tap extraction, as now there is a high degree of uncertainty in the Fourier space of the signal. A Gaussian window is then used to remove the Fresnel ripples in the Fourier space shown by Figure S3(c). Therefore, processing with a Gaussian window allows us to accurately extract tap delays on arbitrary wavelength bandwidths ranging from 10 nm as shown in the main article, and $\sim$ 100 nm as shown here. This process works for both cases in the 4-tap and 8-tap designs as shown by Figure S3(d), Figure S3(e), and Figure S3(f). However, accurate tap extraction is not just about obtaining the resulting delay lines in Fourier space. We also need to assess how the addition of each delay line results in a highly correlated representation of $G(\omega)$ in the wavelength space. We address this in the following section.

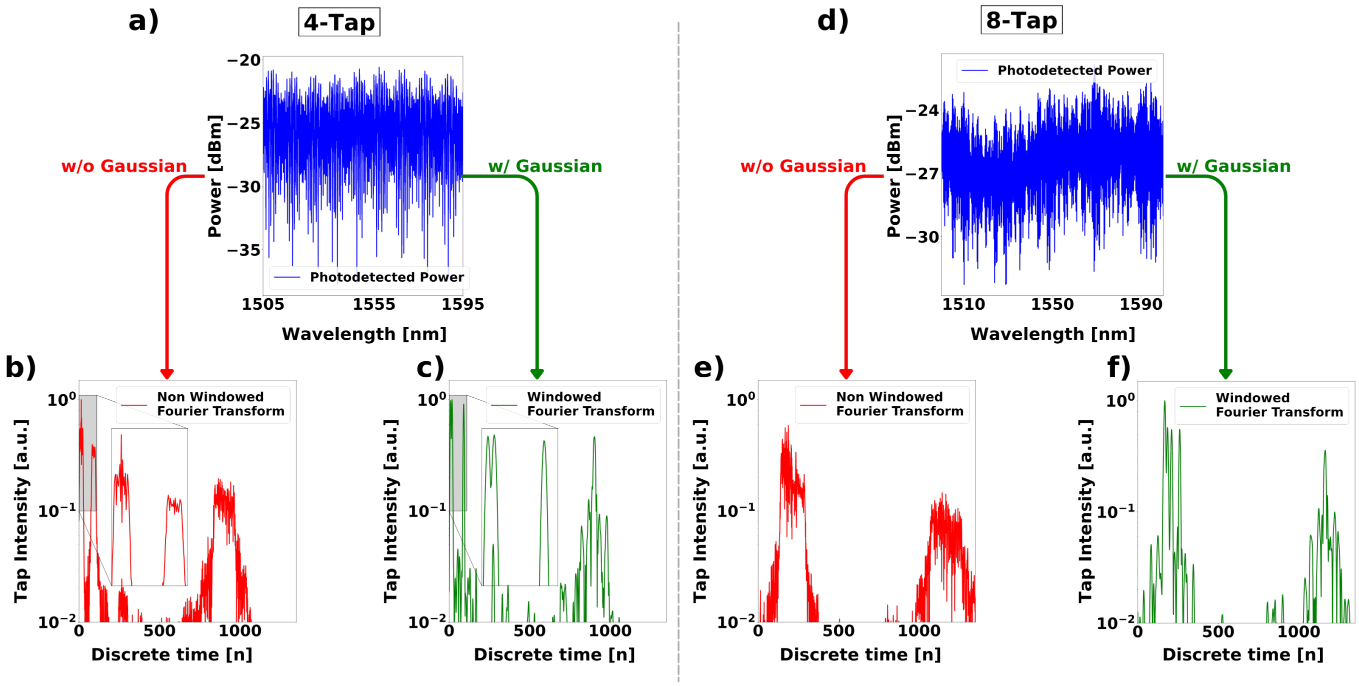

Figure S3. **Dispersion Management Strategies for Large ΔL.** Data for a $\sim$ 100 nm bandwidth showing the (a) 4-tap and (d) 8-tap circuits described in the main article. The phase retrieval process is performed as described, and the $G(\omega)$ Fourier transform is shown for the (b) 4-tap and (e) 8-tap circuits, showcasing the resulting Fresnel ripples owed to dispersion. (c, f) Processing with a Gaussian window removes the Fresnel ripples, uncovering the delay lines present in the circuits.

B. Signal Reconstruction

Proper signal conditioning for its Fourier space processing is essential when dealing with larger bandwidths. However, the information conveyed in this space might not be an accurate representation of the multiple delay paths present in the MZI structure. While delay taps are uncovered by the Gaussian window process shown in Figure S3(f), we ought to assess how well correlated these taps are with the original signal, and whether or not these are an accurate model for the original signal. To do so, we create two models based on the extracted taps. The first is based on Fourier transform information, whereas the second is based on the phase model presented in the main article.

The Fourier transform model is given by

$$\hat{G}(\omega) = \sum_{n=1}^{N} |g_n| e^{j f_n k - \arg(g_n)} \tag{11}$$

where g_n corresponds to the n-th Fourier transform peak complex-valued data, f_n is the frequency derived from the peak location as $2\pi N_{peak}/N$ where N_{peak} is the peak's index location, and N is the signal length. Finally, k is a regression vector going from 0 to N in integer steps. The phase model is the same model previously described in the main article, and it is given by the second-degree polynomial regression on Δf.

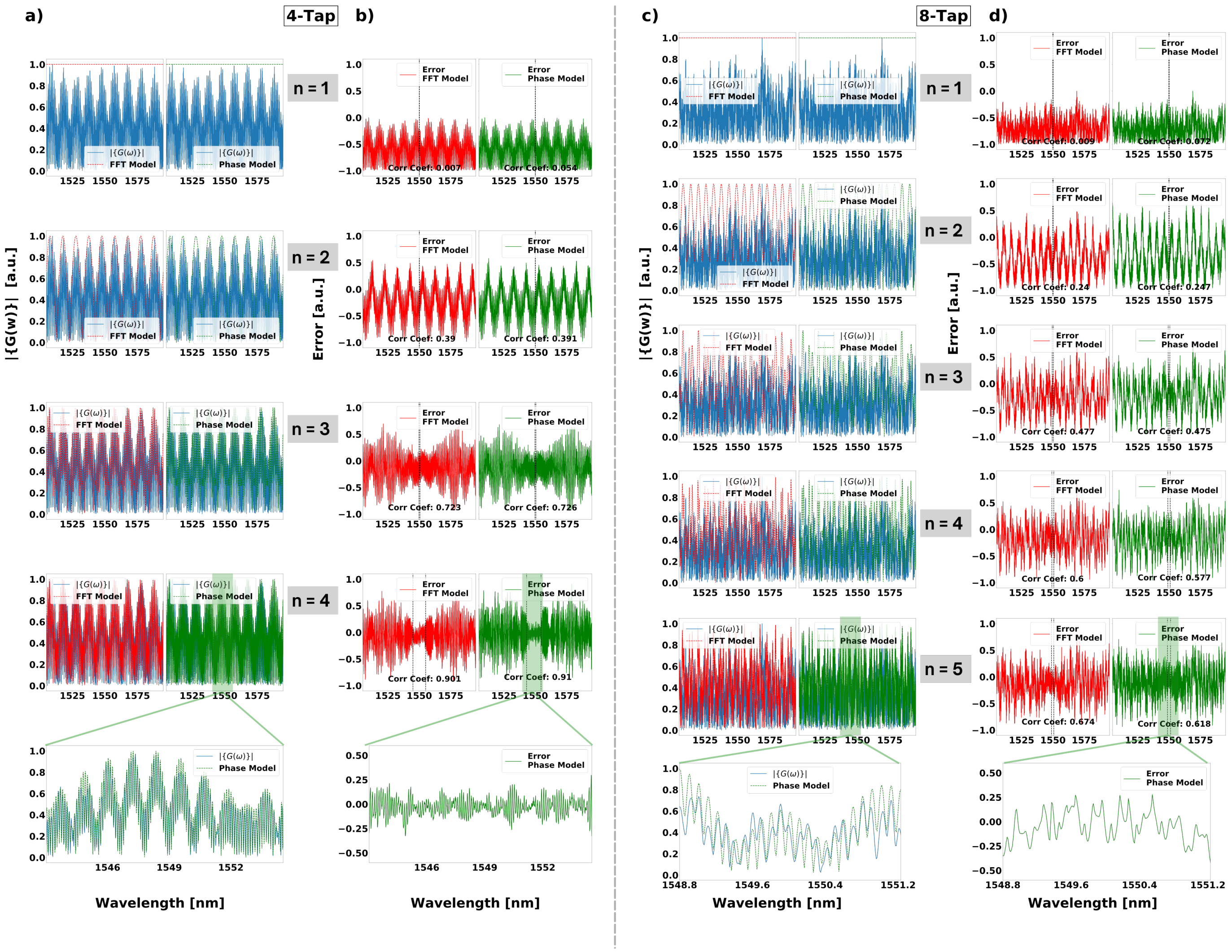

Figure S4. **Signal ($G(\omega)$) Reconstruction for Valid Bandwidth Extraction for Both 4 and 8 tap multi-ΔL Designs**. This figure demonstrates that the selection of sparse taps is key for accurate signal reconstruction. Specifically, the signal reconstruction process is shown for the 4 and 8-tap circuits described in the article. (a, c) The original signal (blue trace) is compared to the phase model (green trace) described in the main article and the FFT-based model (red trace) as we add individual n-th signal elements. (b, d) The error and correlation coefficient of the regression within the valid bandwidth are shown, and for the 4-tap case, the final correlation coefficient values of ~0.9 and ~0.91 for the FFT and phase models, respectively. In contrast, the 8-tap is not properly reconstructed due to improper sparse tap ratio configuration.

As shown in Figure S4(a), as individual signal components are added to the model, the regression begins to resemble the original signal $G(\omega)$, as evidenced by the error and correlation coefficient in Figure S4(b). In contrast, we infer that the peaks obtained from the 8-tap design do not accurately represent $G(\omega)$ as the regression algorithm does not converge into $G(\omega)$ for this case, as shown in Figure S4(c). As a result, this demonstrates that in addition to proper tap separability in the Fourier space, we ought to be careful with the tap ratios, as they must remain integer increases from the smallest $\Delta L1$ as the bandwidth-defining tap. The 8-tap design does not support this condition, which inevitably causes spectral leakage similar to the Fresnel ripples, creating artificial spectral lines that do not correlate with the original signal. This process is demonstrated in the error figures and correlation coefficients obtained in Figure S4(d). Lastly, in Figure S4(b) we see that the proper bandwidth

of operation where signal reconstruction is valid is from 1545 nm to 1555 nm where the model and the obtained $G(\omega)$ data are highly correlated (correlation coefficient ~ 0.91). This is the bandwidth presented in the original paper, and deviations from this bandwidth compromise the phase recovery algorithm presented therein. We have successfully demonstrated that this approach is limited by the directional couplers' bandwidth, as demonstrated by the results of this regression. Finally, we demonstrate that this can be used for in-situ characterization of photonic integrated circuits via the use of a singular interferometric circuit, and this phase-retrieval process by proper dispersion handling and tap design.

IV. Wavelength Model: Linear vs. second order

A second-degree polynomial model was employed to represent the phase as a function of wavelength (or frequency) in Fig. 3 of the main article. A linear model would introduce bias into the error, resulting in a higher RMSE, indicating that the signal is not adequately modeled. As illustrated in Figure S5, a linear approximation is sufficient for delay paths where dispersion is negligible (FSR > 1 nm). However, for larger delays such as ΔL_3 and ΔL_4, dispersion becomes significant, leading to systematic error bias. Consequently, a linear model cannot capture all signal components when ΔL is sufficiently large, necessitating the use of a second-order polynomial fit

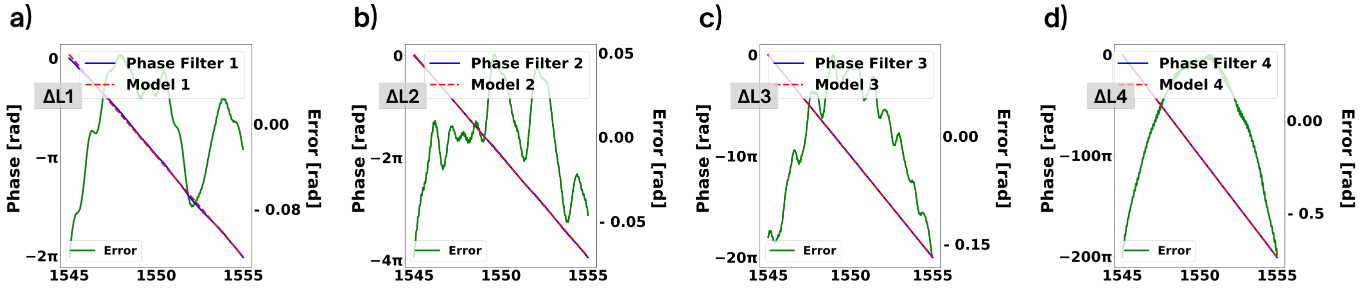

Figure S5. **Linear Phase Model for multi-ΔL Design**. Regression results for a linear phase model for (a) $\Delta L1$, (b) $\Delta L2$, (c) $\Delta L3$, and (d) $\Delta L4$, where dispersion becomes evident at $FSR \leq 1\ nm$ as seen by the parabolic dependence on the error.

V. Application: Complex Fourier Transform Spectroscopy

Here, we extend the work presented in the main paper to the broader application of computational spectroscopy. Specifically, here we present how the minimum-phase design framework enables complex-valued Fourier-transform spectroscopy for reconstructing arbitrary spectra in a single-stream circuit only from intensity measurements. This approach overcomes the manufacturing sensitivity of conventional FTS, which has hindered reconstruction robustness [4]. In contrast, the minimum-phase approach directly fits the spectral phase function, thereby absorbing manufacturing variations in our calibration procedure. Furthermore, owing to the complex-valued nature of our approach, we perform interferogram alignment by leveraging phase slope multiplication in the complex domain, enabling lossless interpolation. In conjunction, these features allow the reconstruction of both the amplitude and the phase of broadband sources.

As shown in Fig. S6(a), the experimental setup closely follows that of Figure 3 in the main article. The minimum-phase MZI circuit was designed and fabricated using the Applied Nanotools SiN MPW process. It includes four ΔL values: 121 μm, 242 μm, 1210 μm, and 12103 μm, corresponding to free spectral ranges (FSRs) of 10, 5, 1, and 0.1 nm, respectively. Edge couplers were aligned using a Maple Leaf automated test setup with a polarization-maintaining fiber array. Chip characterization employed a Keysight 81606A tunable laser and N7744A photodetector, sweeping from 1500–1600 nm in 1 pm steps; analysis was limited to the 1545–1555 nm range. Thermo-optic tuning was achieved using DC probes and a Keithley 2400 SMU sourcing 0–10 V in 0.5 V increments, with a wavelength sweep at each voltage.

Leveraging the circuit's minimum-phase property, the phase response is retrieved from the measured intensity via a Hilbert transform (Fig. S6(b,c)). Subsequent digital filtering around each delay line isolates the individual ΔL interferometers (Fig. S6(d)). The filtered signals reveal the spectral phase response as a function of the electrical power applied to each phase shifter (Fig. S6(e–h)). This experimental phase data enables direct characterization of the optical path delay sweep, $\Delta L(P)$, which is then modeled using a polynomial fit (Fig. S6). Using this delay model, the interferograms are aligned by applying the phase response expressed as $\propto e^{\beta \Delta L_i(P)}$ (Fig. S6(m–p)). Finally, the Fourier transform of the averaged aligned interferograms yields the reconstructed spectrum.

The reconstruction algorithm is evaluated across four scenarios: calibration, a chirped Gaussian, a four-arm asymmetric Mach–Zehnder interferometer (MZI), and a micro-ring resonator (Fig. S7). The calibration case involves sweeping a laser from 1545 nm to 1555 nm (Fig. S7(a–c)), corresponding to a zero-phase flat spectrum. This step enables characterization of spectrometer-induced biases and normalization of systematic errors before testing simulated signals. The remaining three cases demonstrate reconstruction of complex spectra through simulation (Fig. S7(d–l)). We highlight that the calibration results are derived from experimental data obtained on the same photonic chip presented in Figure 3 from the main article, whereas the

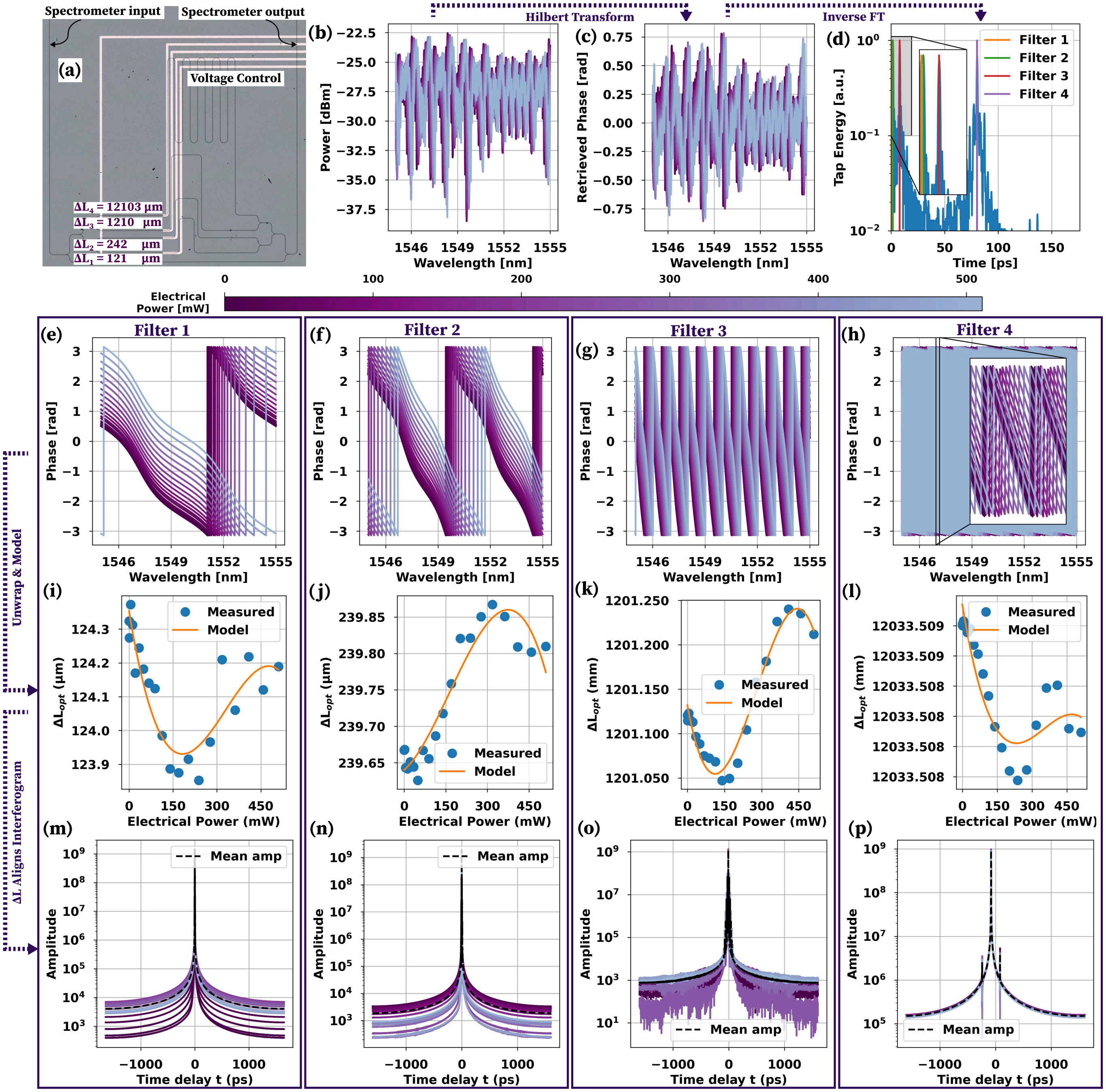

Figure S6. **Spectrometer Experimental Calibration**. (a) Experimental setup for gathering the spectrometer calibration data using a Keysight tunable laser and PD combo with a Keithley 2400 Source-Measure-Unit (SMU). (b) Photodetected power and (c) retrieved phase using the Hilbert transform and leveraging the minimum-phase design of the resulting MZI system. (d) The inverse Fourier Transform of the constructed complex-valued response allows us to filter each ΔL delay line for further post-processing. The application of each filter provides the phase of each ΔL_i (e-h). Subsequently, each spectral phase is fitted using a linear model to obtain $\Delta L(P)$ (i-l). The data from this is then used to align each interferogram by using the phase factor $\propto \Delta L(P)/c$ at each electrical power value (m-p).

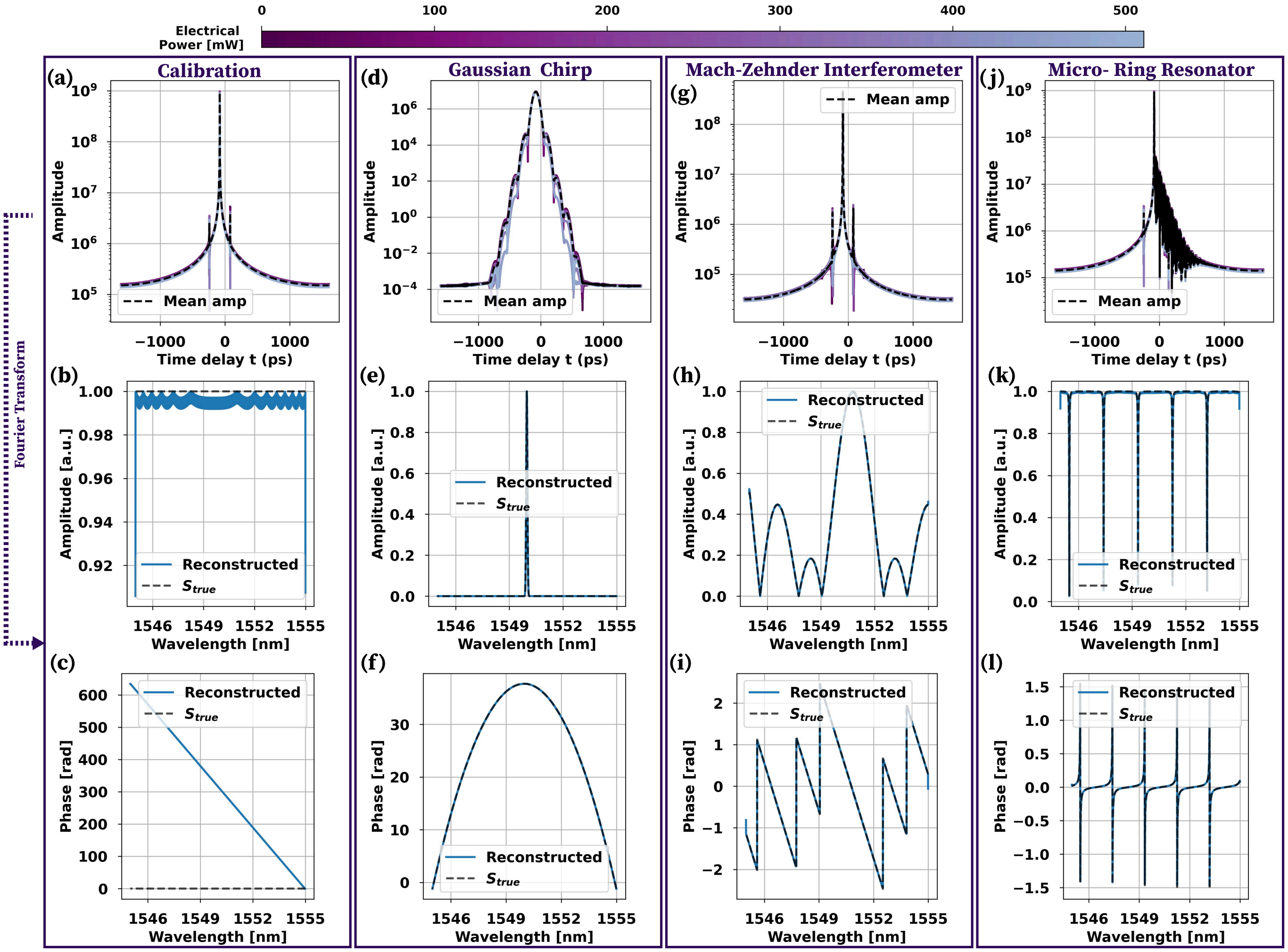

Figure S7. **Reconstruction of Simulated Signals**. (a) Interferogram, (b) magnitude, and (c) phase reconstruction of the calibration data for Filter 4. This is used as a calibration step, where the phase and magnitude serve as normalization factors for simulating synthetic data. (d) Interferogram, (e) magnitude, and (f) phase reconstruction of a chirped Gaussian. (g) Interferogram, (h) magnitude, and (i) phase reconstruction of a 4-arm (3 optical path differences) MZI. (j) Interferogram, (k) magnitude, and (l) phase reconstruction of a micro-ring resonator. We observe proper reconstruction of all simulated signals using the data from Filter 4.

other cases represent simulated reconstructions. Future work will focus on experimental validation of these complex spectra. Due to the minimum-phase design framework, the method supports accurate reconstruction of diverse signal profiles. This capability paves the way for complex-valued spectroscopy.

VI. Signal-to-Noise Ratio Analysis for Multi-Tap Interferometer

We derive the per-tap signal-to-noise ratio (SNR) for the multi-tap interferometer architecture shown in Section II.C of the main Article. The input optical power is denoted by P_{in}. After the initial 50:50 splitter, the optical fields in the reference arm and the nth interferometric tap are written as

$$E_r = A_r e^{i\phi_r}, \qquad E_n = A_n e^{i\phi_n}, \tag{12}$$

where the field amplitudes and phases are given by

$$A_r = \sqrt{\frac{P_{\text{in}}}{2}}\, e^{-\alpha \frac{L_r}{2}}, \qquad \phi_r = \beta L_r, \tag{13}$$

$$A_n = \sqrt{\frac{P_{\text{in}}}{2}}\, e^{-\alpha \frac{L_n}{2}}, \qquad \phi_n = \beta L_n. \tag{14}$$

Here, L_r and L_n denote the reference and nth tap path lengths, respectively, α is the loss, and $\beta = 2\pi n_{\text{eff}}/\lambda$ is the propagation constant. The field at the interferometer output is given by

$$E_{\text{out}} = E_r + \sum_{n=1}^{N} E_n, \tag{15}$$

leading to a detected optical power

$$P_{\text{det}} = |E_{\text{out}}|^2 = |E_r|^2 + \sum_n |E_n|^2 + 2\sum_n \text{Re}[E_r^* E_n] + 2\sum_{m\neq n} \text{Re}[E_m^* E_n]\,. \tag{16}$$

Under the minimum-phase operating condition, the reference-arm power dominates the total optical power,

$$|E_r| \gg \sum_n |E_n|, \tag{17}$$

so that both tap self-beating terms and nth-tap-to-mth-tap beating terms are negligible. The detected power may therefore be approximated as

$$P_{\text{det}} \approx A_r^2 + 2\sum_n A_r A_n \cos(\phi_n - \phi_r)\,. \tag{18}$$

The interferometric contribution associated with the nth tap is thus given by

$$S_n = 2A_r A_n \cos(\Delta\phi_n) = 2\sqrt{P_r P_n}\cos(\Delta\phi_n)\,, \tag{19}$$

where $P_r = A_r^2$, $P_n = A_n^2$, and $\Delta\phi_n = \phi_n - \phi_r$.

We derive the full signal-to-noise model, including both amplitude fluctuations of the detected optical powers and phase fluctuations arising from thermo-optic and environmental drift. Starting with the amplitude variations, Small fluctuations in the reference and tap powers, $P_r \to P_r + \delta P_r, P_n \to P_n + \delta P_n$, lead to a first-order variation

$$\delta S_n^{(\text{amp})} = \frac{\partial S_n}{\partial P_r}\delta P_r + \frac{\partial S_n}{\partial P_n}\delta P_n = \sqrt{\frac{P_n}{P_r}}\,\delta P_r + \sqrt{\frac{P_r}{P_n}}\,\delta P_n. \tag{20}$$

Assuming the amplitude fluctuations δP_r and δP_n are uncorrelated,

$$\sigma^2_{S_n,\text{amp}} = \frac{P_n}{P_r}\,\sigma^2_{P_r} + \frac{P_r}{P_n}\,\sigma^2_{P_n}, \tag{21}$$

The σ_{P_r} and σ_{P_n} values are taken directly from the detector noise specification (Keysight N7744a detector). These values incorporate detector shot noise, TIA noise, ADC noise, internal bandwidth filtering, and range-dependent gain, therefore allowing us to encapsulate all noise sources related to photodetection in this model.

We model the phase noise by letting the reference and tap accumulate zero-mean phase fluctuations $\delta\phi_r, \delta\phi_n$, giving a relative phase fluctuation $\delta(\Delta\phi_n) = \delta\phi_n - \delta\phi_r$ with variance $\sigma^2_{\Delta\phi} = \sigma^2_{\phi_n} + \sigma^2_{\phi_r}$. Linearizing the cosine term in (19),

$$\cos(\Delta\phi_n + \delta\Delta\phi) \approx \cos\Delta\phi_n - \sin\Delta\phi_n\, \delta(\Delta\phi), \tag{22}$$

so that

$$\delta S_n^{(\text{phase})} = -2\sqrt{P_r P_n}\,\sin(\Delta\phi_n)\,\delta(\Delta\phi_n). \tag{23}$$

Hence, the phase-to-power noise variance is

$$\sigma^2_{S_n,\text{phase}} = 4P_r P_n\,\sin^2(\Delta\phi_n)\,\sigma^2_{\Delta\phi}. \tag{24}$$

Phase fluctuations arise from thermo-optic index noise, mechanical, and environmental drift. Since amplitude and phase noise originate from independent physical processes, their variances add:

$$\sigma_{S_n}^2 = \sigma_{S_n,\mathrm{amp}}^2 + \sigma_{S_n,\mathrm{phase}}^2. \tag{25}$$

Substituting (21) and (24),

$$\boxed{\sigma_{S_n}^2 = \frac{P_n}{P_r}\,\sigma_{P_r}^2 + \frac{P_r}{P_n}\,\sigma_{P_n}^2 + 4P_rP_n\,\sin^2(\Delta\phi_n)\,\sigma_{\Delta\phi}^2.} \tag{26}$$

Using S_n from Eq. (19) and noise variance (26), the per-tap SNR is

$$\boxed{\mathrm{SNR}_n = \frac{4P_rP_n\,\cos^2(\Delta\phi_n)}{\frac{P_n}{P_r}\,\sigma_{P_r}^2 + \frac{P_r}{P_n}\,\sigma_{P_n}^2 + 4P_rP_n\,\sin^2(\Delta\phi_n)\,\sigma_{\Delta\phi}^2}.} \tag{27}$$

Equation (27) cleanly separates amplitude noise, which is set by the detector specifications and optical power levels, from phase noise, which grows with the interferometric path-length imbalance and dominates for long delay lines. We use this expression to evaluate the maximum length that can be recovered in this minimum-phase multi-tap interferometer architecture. Notably, the wavelength regression algorithm depends on resolving accurate delay lines in the interferogram Fourier space. As a result, the $\mathrm{SNR_n}$ value provides an upper bound on the tap length before it becomes buried within the noise levels.

A. Detector-Limited Maximum Tap Length

We first evaluate the maximum usable delay length $L_n^{\max}$ under the assumption that detector noise dominates the interferometric degradation. In this regime, the variance of the interferometric signal is given solely by the amplitude-noise contribution in Eq. (26). With phase noise neglected, the per-tap SNR from Eq. (27) reduces to

$$\mathrm{SNR}_n^{(\mathrm{amp})} = \frac{4P_rP_n}{\frac{P_n}{P_r}\,\sigma_{P_r}^2 + \frac{P_r}{P_n}\,\sigma_{P_n}^2}. \tag{28}$$

Under the minimum-phase operating condition $P_r \gg P_n$, the second term in the denominator dominates, giving the approximation

$$\mathrm{SNR}_n^{(\mathrm{amp})} \approx \frac{4P_rP_n}{(P_r/P_n)\sigma_P^2} = \frac{4P_n^2}{\sigma_P^2}, \tag{29}$$

where we have set $\sigma_{P_r} = \sigma_{P_n} = \sigma_P$, appropriate for a single detector channel and a fixed power-measurement range. Solving $\mathrm{SNR}_n^{(\mathrm{amp})} \geq \mathrm{SNR}^\star$ yields a minimum tap power

$$P_{n,\min} = \frac{\sqrt{\mathrm{SNR}^\star}}{2}\,\sigma_P. \tag{30}$$

Using the tap-loss relation $P_n = \frac{P_{\mathrm{in}}}{2}\,e^{-\alpha L_n}$, the detector-noise-limited maximum delay length follows from $P_n = P_{n,\min}$:

$$\boxed{L_n^{\max}(\mathrm{amp}) = \frac{1}{\alpha}\,\ln\!\left(\frac{P_{\mathrm{in}}}{\sigma_P\sqrt{\mathrm{SNR}^\star}}\right).} \tag{31}$$

This expression directly incorporates the detector noise from the manufacturer's datasheet σ_P and therefore provides an accurate detector-noise–limited bound for the usable delay length of each tap. As shown in the main analysis, using the manufacturers (Keysight N7744a) detector-noise values (rescaled to the used 200 μs integration time) yields amplitude-limited maximum delays in the range $L_n^{\max}(\mathrm{amp}) \approx 55$–$66$ cm for $\mathrm{SNR}^\star = 10$–20 and detector noise levels of $\sigma_P = 88pW$–$890pW$ from the datasheet information.

B. Phase-Noise-Limited Maximum Tap Length

We now evaluate the maximum usable delay length under the assumption that phase noise dominates the interferometric degradation. Thus, we consider the variance of the nth tap signal is given solely by the phase-noise contribution in Eq. (26). With amplitude noise neglected, the per-tap SNR from Eq. (27) reduces to

$$\mathrm{SNR}_n^{(\phi)} = \frac{4P_rP_n\cos^2(\Delta\phi_n)}{4P_rP_n\sin^2(\Delta\phi_n)\,\sigma_{\Delta\phi}^2} = \frac{\cot^2(\Delta\phi_n)}{\sigma_{\Delta\phi}^2}. \tag{32}$$

The interferometer is wavelength-swept, and we are looking for the longest allowable delay tap (smallest FSR). As a result, the cos and sin terms will average over the wavelength frame, $\sin\Delta\phi_n \approx \cos\Delta\phi_n \approx 1/\sqrt{2}$, yielding the approximation

$$\mathrm{SNR}_n^{(\phi)} \approx \frac{1}{\sigma_{\Delta\phi}^2}. \tag{33}$$

Thus, in the phase-noise-limited regime, the per-tap SNR is independent of optical power. Using a simple thermo-optic model as a first-order approximation, we let $\xi = dn_{\text{eff}}/dT$ denote the effective thermo-optic coefficient of the guided mode and let σ_T denote the RMS temperature fluctuation over the acquisition window. The accumulated phase fluctuation is

$$\sigma_{\Delta\phi}(L) \approx \frac{2\pi}{\lambda}\,\xi\,L\,\sigma_T. \tag{34}$$

Substituting this into Eq. (33) and solving for $\text{SNR}_n^{(\phi)} \geq \text{SNR}^\star$ yields

$$\boxed{L_n^{\max}(\phi) = \frac{\lambda}{2\pi\,\xi\,\sigma_T\,\sqrt{\text{SNR}^\star}}.} \tag{35}$$

This expression directly incorporates the thermo-optic coefficient of the waveguide material and the temperature fluctuations over the acquisition window, thereby providing a physically grounded phase-noise–limited bound for the usable delay of each tap. using the values of $\xi = 2.5 \times 10^{-5}\ \text{K}^{-1}$ [5] for SiN at $\lambda = 1550\ \text{nm}$ and a target SNR of $\text{SNR}^\star = 10$, the thermo-optic phase-noise limit spans $L_n^{\max}(\phi) \approx 2\ \text{cm}$ to $2\ \text{m}$ for temperature fluctuations in the range $\sigma_T = 100\ \text{mK}$ to $10\ \text{mK}$. Under typical ambient conditions, the phase-noise–limited maximum usable delay is therefore $L_n^{\max}(\phi) \approx$ 2–20 cm, substantially smaller than the detector-noise-limited bound and the dominant constraint in practical multi-tap interferometric PICs.

These calculations show that SNR is not the biggest constraint on the maximum usable delay. Increasing the path length decreases the free spectral range (FSR) accordingly, increasing the demands on the wavelength stepping resolution of the calibration laser. As a result, for delay lengths that approach this SNR-limited bound, the FSR reaches the picometer scal,e placing impractical constraints on the laser tuning capabilities. While increasing the delay length improves the wavemeter resolutio,n as shown in the main article, it simultaneously imposes stricter requirements on calibration spectral resolution. Therefore, these calculations demonstrate that the practical performance of the wavelength regression method is primarily determined by system-level constraints, rather than the phase-retrieval process itself.

References

[1] J. Bechhoefer, "Kramers–kronig, bode, and the meaning of zero," *American Journal of Physics*, vol. 79, no. 10, pp. 1053–1059, Oct 2011. [Online]. Available: https://doi.org/10.1119/1.3614039

[2] M. M. Seron, J. H. Braslavsky, and G. C. Goodwin, *Fundamental Limitations in Filtering and Control*, ser. Communications and Control Engineering, B. W. Dickinson, A. Fettweis, J. L. Massey, J. W. Modestino, E. D. Sontag, and M. Thoma, Eds. London: Springer London, 1997. [Online]. Available: http://link.springer.com/10.1007/978-1-4471-0965-5

[3] X. Xu, G. Ren, T. Feleppa, X. Liu, A. Boes, A. Mitchell, and A. J. Lowery, "Self-calibrating programmable photonic integrated circuits," *Nature Photonics*, vol. 16, no. 8, pp. 595–602, Aug 2022. [Online]. Available: https://www.nature.com/articles/s41566-022-01020-z

[4] A. Li, J. Davis, A. Grieco, N. Alshamrani, and Y. Fainman, "Fabrication-tolerant fourier transform spectrometer on silicon with broad bandwidth and high resolution," *Photonics Research*, vol. 8, no. 2, pp. 219–224, Feb 2020. [Online]. Available: https://opg.optica.org/prj/abstract.cfm?uri=prj-8-2-219

[5] A. Arbabi and L. L. Goddard, "Measurements of the refractive indices and thermo-optic coefficients of si3n4 and siox using microring resonances," *Opt. Lett.*, vol. 38, no. 19, pp. 3878–3881, Oct 2013. [Online]. Available: https://opg.optica.org/ol/abstract.cfm?URI=ol-38-19-3878
